# Successful recovery from a subclavicular ulcer caused by lenvatinib for thyroid cancer: a case report

**DOI:** 10.1186/s12957-017-1096-5

**Published:** 2017-01-14

**Authors:** Morimasa Kitamura, Tomomasa Hayashi, Chiaki Suzuki, Shigeru Hirano, Ichiro Tateya, Yo Kishimoto, Koichi Omori

**Affiliations:** 1 Department of Otolaryngology, Head and Neck Surgery, Graduate School of Medicine, Kyoto University, Sakyo-ku, Kyoto, 606-8507 Japan; 2 Department of Clinical Oncology, Graduate School of Medicine, Kyoto University, Kyoto, Japan; 3 Department of Otolaryngology-Head and Neck Surgery, Kyoto Prefectural University of Medicine, Kyoto, Japan

**Keywords:** Lenvatinib, Sorafenib, Antiangiogenic tyrosine kinase inhibitor, Thyroid cancer, Surgery

## Abstract

**Background:**

There are currently no effective therapeutic methods for locally recurrent, metastatic, or progressive radioactive iodine (RAI)-refractory differentiated thyroid cancer. However, multitargeted tyrosine kinase inhibitors (TKIs) such as lenvatinib or sorafenib have been approved for patients with RAI-refractory differentiated thyroid cancer as a second targeted therapy, and these agents can prolong patient survival. However, several cases have been reported that TKIs have caused fatal complications such as fistula formation or bleeding.

**Case presentation:**

We report a case of a 53-year-old woman, who underwent repeated neck dissections and RAI therapy after total thyroidectomy in an outside hospital. Pathology revealed a papillary carcinoma of the tall cell variant. Locoregional recurrence was not under control; therefore, she visited our hospital. Although surgery was performed for locoregional recurrences three times in our hospital, they were not under control and distant metastases were found in the lung and bone a year later. Therefore, although sorafenib was initiated, the locoregional recurrence progressed 6 months later and computed tomography (CT) showed a 7-cm mass in the right subclavicular lesion. Lenvatinib was started at a dose of 24 mg daily. However, although tumor was rapidly reduced, an ulcer occurred in the right subclavicular lesion and was gradually increasing in size. The pulsation of subclavicular artery was found in the deep portion of the ulcer. Therefore, a pectoralis major myocutaneous flap was transplanted to cover the ulcer. Lenvatinib was an antiangiogetic TKI; therefore, it was preoperatively discontinued for 8 days and postoperatively for 12 days. The postoperative course was uneventful.

**Conclusions:**

Fistula formation or bleeding is known to be a severe side effect of antiangiogenic TKIs such as lenvatinib or sorafenib. There is a possibility that severe complications can occur when initiating TKIs in patients whose tumor has invaded into the skin, vessels, trachea, esophagus, and other areas. Therefore, it is necessary to use antiangiogenic TKIs very carefully. It is important to determine the appropriate time to start TKIs; however, there is no established protocol for this, and it is a problem that needs urgent attention.

## Background

Thyroid cancer is a common malignant tumor that has a good prognosis. However, we sometimes encounter patients with locally recurrent, metastatic, or progressive radioactive iodine (RAI)-refractory differentiated thyroid cancer. Treatment options are limited in these cases. A multitargeted tyrosine kinase inhibitor (TKI) such as lenvatinib or sorafenib was recently approved as a second targeted therapy for these cases. Treatment of advanced forms of endocrine cancer which are not responsive to cytotoxic chemotherapies is challenging, and use of TKIs is gaining a growing role in this clinical context [1].

Lenvatinib or sorafenib is indicated for the treatment of locally recurrent, metastatic, or progressive RAI-refractory differentiated thyroid cancers. The approval of lenvatinib was based on the results of the randomized, double-blind, multinational, phase 3 SELECT study, in which lenvatinib significantly improved median progression-free survival (PFS) and overall response rate compared with placebo in patients with locally recurrent, metastatic, or progressive RAI-refractory differentiated thyroid cancers [2].

Lenvatinib is a multitargeted TKI of vascular endothelial growth factor receptors 1–3 (VEGFRs 1–3), fibroblast growth factor receptors 1–4 (FGFRs 1–4), platelet-derived growth factor receptor α (PDGFR-α), and the rearranged during transfection (*RET*) and c-KIT signaling networks, which are implicated in pathogenic angiogenesis, tumor growth, and cancer progression [3].

Sorafenib significantly prolonged median PFS relative to placebo in the randomized, double-blind, multinational, phase 3 DECISION study [4]. Sorafenib is a multitargeted TKI of VEGFR 1–3, PDGF-β, c-KIT, FMS-like tyrosine kinase-3, and RAF kinases [5].

TKIs that block VEGFR, such as lenvatinib or sorafenib, are known to cause delayed healing. Severe complications of lenvatinib such as bleeding or fistula formation have been observed in the post-approval 6 months study in Japan [6]. Surgery is not generally performed in these cases because wound healing is delayed when a TKI of VEGFR is being used. TKI has to be withdrawn preoperatively if surgery needs to be performed. However, when TKI is withdrawn, tumor size can increase rapidly, and it is dangerous to withdraw it for a long time. We initiated lenvatinib for a patient with locally recurrent and metastatic progressive RAI-refractory differentiated thyroid cancer, and taking lenvatinib caused the formation of a giant ulcer in the right subclavicular lesion.

We report that we encountered a severe case of a subclavicular ulcer caused by lenvatinib and successfully avoided rupture of the subclavicular artery or vein by surgically protecting the major vessels with a pectoralis major musculocutaneous (PMMC) flap.

## Case presentation

A 53-year-old woman underwent repeated neck dissections after undergoing total thyroidectomy and was given a total of 200 mCi (100 mCi twice) RAI therapy in an outside hospital. Pathology revealed a papillary carcinoma of the tall cell variant. Locoregional recurrence was not under control; therefore, she visited our hospital for a second opinion regarding a treatment strategy.

Surgery was performed for locoregional recurrences three times in our hospital, but it was not under control and distant metastases were found in the lung and iliac bone a year later. Therefore, sorafenib was initiated at a dose of 800 mg daily and was gradually reduced because of side effects, resulting in a continuous dose of 400 mg. The locoregional recurrence and distant metastases progressed 6 months later. We waited for lenvatinib to be approved in Japan and then administered it.

**Fig. 1:**
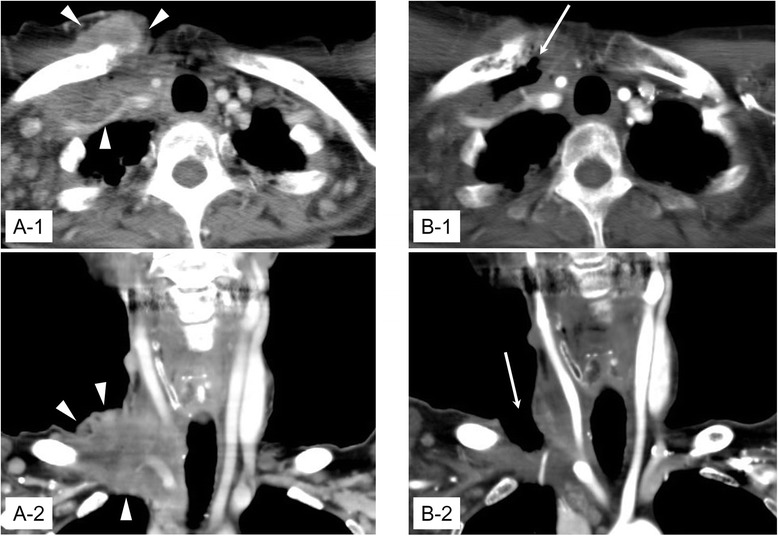
**A-1, A-2** Enhanced CT findings before starting lenvatinib. Tumor is invaded into subcutaneous tissue in the right subclavicular lesion (*arrowhead*). **B-1, B-2** Enhanced CT findings just before surgery (day 32). A large ulcer occurred in right subclavicular lesion (*arrow*)

**Fig. 2:**
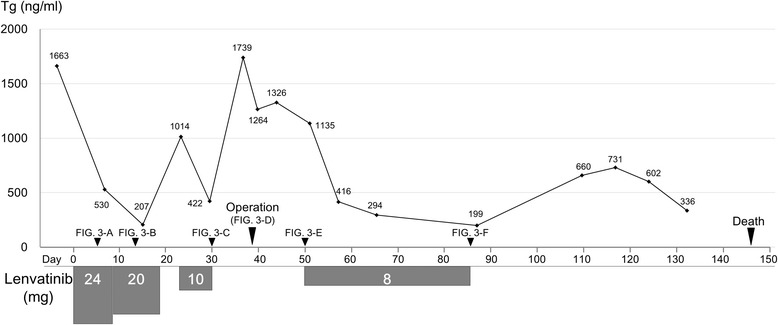
A change of thyroglobulin (Tg). The level of Tg is rapidly decreasing when taking lenvatinib

Before lenvatinib was initiated, computed tomography (CT) showed a 7-cm mass in the right subclavicular lesion (Fig. 1A-1, A-2), multiple affected lymph nodes in the bilateral axilla, and multiple nodules in the lung and iliac bone. Her thyroglobulin (Tg) was 1663 ng/mL (Fig. 2), and her thyroid-stimulating hormone was 0.027 μIU/mL.

**Fig. 3:**
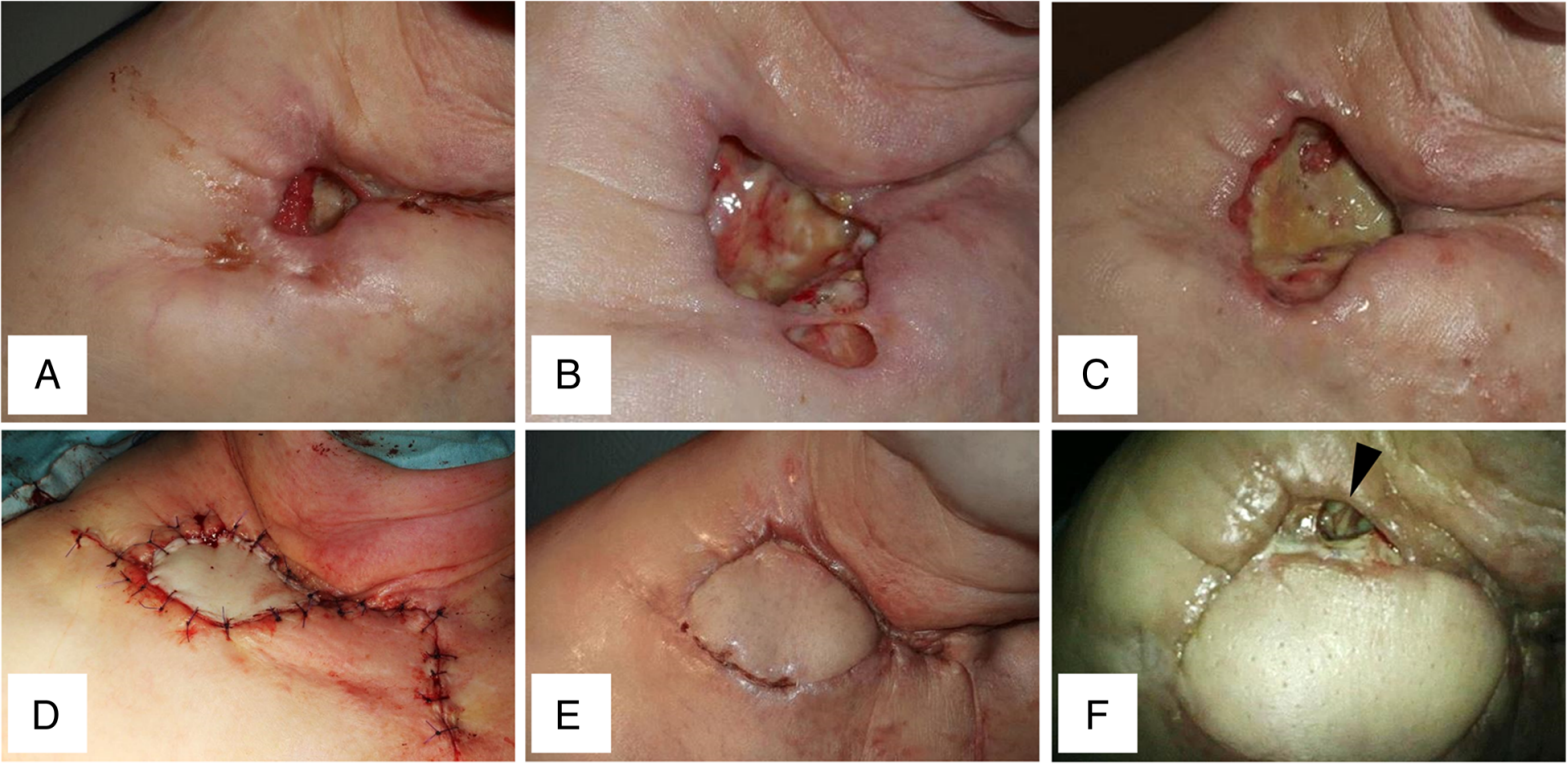
Gross findings in the right subclavicular lesion. **a** Day 5. A small ulcer was found. **b** Day 13. The size of ulcer was gradually increased. **c** Day 30. The ulcer had become larger. **d** Day 38, operation. PMMC flap was transplanted. **e** Day 50/POD 12. The flap was engrafted well. **f** Day 68/POD 30. A fistula was found at the posterior side of flap (*arrowhead*)

After lenvatinib was initiated at a dose of 24 mg daily, her Tg decreased rapidly (Fig. 2). An ulcer occurred in her right subclavicular lesion at the same time and was gradually increasing in size (Fig. 3a, b). On day 8, the lenvatinib was reduced to a dose of 20 mg. The lenvatinib was cancelled on day 18 because a pulsation of the right subclavicular artery was found in the deep portion of the ulcer. Tg rapidly increased as soon as the lenvatinib was cancelled (Fig. 2). Therefore, 10 mg of lenvatinib was started on day 23. Tg gradually decreased again, but the size of the ulcer did not change. The patient was at risk of bleeding from the right subclavicular artery or vein (Fig. 1B-1, B-2). Therefore, we decided to cover the ulcer using a PMMC flap.

We considered the following three points in the surgical approach:

1. Lenvatinib is an antiangiogenic TKI; therefore, wound healing is delayed. We decided to withdraw it 8 days preoperatively.
2. A large flap was needed to cover the ulcer. Therefore, we selected a PMMC flap.
3. After the wound was confirmed to be firmly engrafted, lenvatinib was re-started at a lower dose.

Lenvatinib was preoperatively discontinued for 8 days, and surgery was performed on day 38 (Fig. 2). The tumor and clavicle were resected as much as possible during the surgery. The margin of the ulcer revealed malignancy on intraoperative pathological examination. The PMMC flap (7 × 5 cm) was transplanted to the ulcer (Fig. 3d).

Her postoperative course was uneventful (Fig. 3e). We confirmed the wound was firmly engrafted and started 8 mg of lenvatinib on day 50 (postoperative day (POD) 12). She was discharged the next day and was followed as an outpatient. A fistula was found on the posterior side of the PMMC on day 86 (POD 48) (Fig. 3f). The tumor had been reduced by the lenvatinib, and a discharge was coming through the fistula. Therefore, the lenvatinib was withdrawn. She then developed dyspnea with right pleural effusion. It was not effective for her to undergo pleurodesis. She died of respiratory failure by lung metastases on day 146 (POD 108).

### Discussion

Antiangiogenic TKIs such as lenvatinib or sorafenib can be used for patients with locally recurrent, metastatic, or progressive RAI-refractory differentiated thyroid cancers. Before TKIs, their only option was symptomatic treatments. It is good news that their treatment options have increased.

Antiangiogenic TKIs such as lenvatinib or sorafenib are known to have mild side effects such as fatigue, skin reactions, hypertension, proteinuria, and delayed healing [7, 8]. However, severe side effects including aerodigestive fistula or bleeding have been reported [9–11]. Therefore, there is a possibility that antiangiogenic TKIs can cause a patient’s early death. It has not been established when they should be started.

The recurrent tumor had infiltrated to the right subclavicular subcutaneous tissue in this case, but lenvatinib was started because of her strong desire to take it. An ulcer formed as expected. The size of the tumor was reduced more than expected, and pulsation of the right supraclavicular artery was found in the ulcer. There was a possibility of a rupture from the subclavian artery or vein in this situation, and it was unsafe to discharge her from the hospital. Therefore, we decided to perform surgery.

Surgery is not performed in general for patients taking TKIs because antiangiogenic TKIs have a side effect of delayed healing. It was a problem as to how long lenvatinib should be withdrawn before and after surgery in this case. The half-life of lenvatinib is about 30 h [12, 13]. The drug is pharmacologically metabolized during a period of approximately five times the half-life. Therefore, most of the lenvatinib should have been metabolized if it was preoperatively withdrawn for more than 7 days. Lenvatinib was preoperatively withdrawn for 8 days in this case. Her postoperative course was uneventful without any complications. Desai et al. recommended that the antiangiogenic TKI, sunitinib, used for the treatment of advanced renal cell carcinoma and gastrointestinal stromal tumors, should be stopped for a minimum of 1 week before surgery (which is approximately four half-lives) and be restarted at least 1 week after surgery [10].

There is no evidence as to when lenvatinib should be restarted postoperatively. After confirming, the wound was engrafted firmly, lenvatinib was restarted on postoperative day 12 at a reduced dose of 8 mg. The patient was discharged the next day.

The wound had been healing well after lenvatinib was restarted, but fistula formation was found on the posterior side of PMMC flap at POD 48. A dead space occurred in the subcutaneous lesion because the tumor was reduced more than expected.

Currently, clinical trials are ongoing in sunitinib and pazopanib and other antiangiogetic TKIs [14, 15], and they are expected to be approved in the future. Tumor reduction can cause serious complications such as bleeding and aerodigestive fistulas in cases of invasion into large vessels, the trachea, and the esophagus. Therefore, it is necessary to make the decision to initiate antiangiogenic TKIs carefully. It was reported by Blevins et al. that antiangiogenic TKIs have caused several cases of serious aerodigestive fistula formation, especially in patients undergoing external beam radiation and in cases with invasion of tumor into the esophagus and trachea; therefore, special attention should be turned towards noticing bleeding or fistula formation when using antiangiogenic TKIs [7]. Once fistula formation occurs, it is necessary to discontinue TKIs. However, tumors tend to rapidly grow during the withdrawal of TKIs. TKIs should be used at a lower dose when restarting.

There is a possibility that initiating antiangiogenic TKIs is accelerating the time of death of patients with locally recurrent, metastatic, or progressive RAI-refractory differentiated thyroid cancer. Therefore, it is very important to determine an appropriate time to start them after considering the patients’ quality of life. However, there is currently no evidence to support this type of decision-making. Therefore, we have to continue to discuss when antiangiogenic TKIs should be initiated.

## Conclusions

Fistula formation or bleeding is known to be a severe side effect of antiangiogenic TKIs such as lenvatinib or sorafenib. There is a possibility that severe complications can occur when initiating TKIs in patients whose tumor has invaded into skin, vessels, trachea, esophagus, and other areas. Therefore, it is necessary to use antiangiogenic TKIs very carefully. It is important to determine the appropriate time to start TKIs; however, there is no established protocol for this, and it is a problem that needs urgent attention.

## Abbreviations

CT: Computed tomography
FGFR: Fibroblast growth factor receptor
PDGFR: Platelet-derived growth factor receptor
PFS: Progression-free survival
PMMC: Pectoralis major musculocutaneous
POD: Postoperative day
RAI: Radioactive iodine
*RET*: Rearranged during transfection
Tg: Thyroglobulin
TKI: Tyrosine kinase inhibitor
VEGFR: Vascular endothelial growth factor receptor
